# Using the length of pleural tag to predetermine pleural invasion by lung adenocarcinomas

**DOI:** 10.3389/fonc.2024.1463568

**Published:** 2024-11-01

**Authors:** Yingdong Chen, Qianwen Huang, Zeyang Lin, Xiaoxi Guo, Yiting Liao, Zhe Li, Anqi Li

**Affiliations:** ^1^ Department of The Radiology, Zhongshan Hospital, Medicine School, Xiamen University, Xiamen, China; ^2^ Department of The Pathology, Zhongshan Hospital, Medicine School, Xiamen University, Xiamen, China; ^3^ Department of The Preventive Health Care, Maternal and Child Health Care Hospital of Jimei District, Xiamen University, Xiamen, China; ^4^ Department of The Thoracic Surgery, Zhongshan Hospital, Medicine School, Xiamen University, Xiamen, China

**Keywords:** spiral CT, pleural invasion, lung adenocarcinomas, iodine uptake, pleural tag, tumor invasiveness

## Abstract

**Introduction:**

Pleural contact is present when the underlying pathology of the pleural tag (PT) involves the pleura. This study aimed to preoperatively predict PI by lung adenocarcinomas (ACCs) with PT, exploring CT imaging parameters indicative of PT consisting of pleura and tumor invasiveness.

**Methods:**

This single-center, retrospective study included 84 consecutive patients diagnosed with solid ACCs with PT, who underwent resection at our hospital between May 2019 and July 2023. CT imaging parameters analyzed included: LPT (the length of PT), defined as the shortest distance from the tumor edge to the retracted pleura. Patients were divided into PI -ve group and PI +ve group according to PI status. Regression analyses were used to determine predictive factors for PI.

**Results:**

The study evaluated 84 patients (mean age, 62.0 ± 13.8 years; 45 females) pathologically diagnosed with ACCs with PT on CT. Multivariate regression analysis identified tumor size (OR 1.18, 95% CI 1.09-1.29, *p* = 0.000), LPT (OR 0.48, 95% CI 0.25-0.91, *p* = 0.03) and multiple PTs to multiple types of pleura (OR 3.58, 95% CI 1.13-11.20, *p* = 0.03) as independent predictors for PI. The combination of these CT features improved the predictive performance for preoperatively identifying PI, achieving high specificity and moderate accuracy. The sensitivity of predicting PI with only LPT < 3 mm was 96.9%.

**Conclusion:**

This study determined that LPT is effective for predetermining PI in ACCs with PT.

## Graphical Abstract

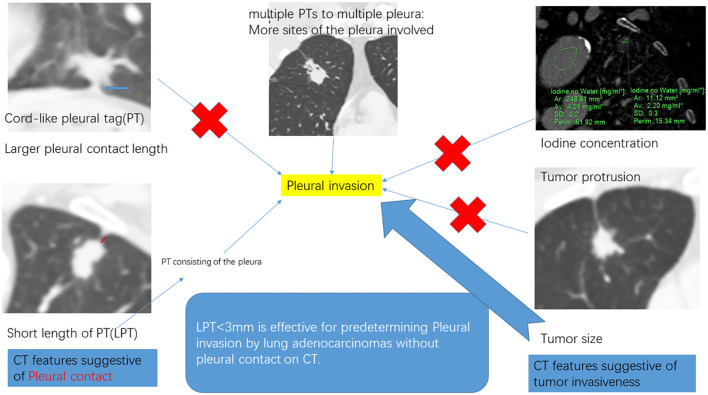

## Introduction

Bronchogenic carcinoma exhibits poor prognosis and high mortality in America and most developed countries (1). Pleural invasion (PI), identified pathologically when the invasion of the bronchogenic carcinomas cells involved the elastic layer of the visceral pleura, correlates with decreased survival rates (2, 3) and influences surgical modality (4). Therefore, preoperative identification of PI status is crucial for clinical decision-making.

Pleural contact observed on CT has been recognized as an independent predictor of PI by lung adenocarcinomas (ACCs) (5–8). However, instances of non-small cell lung cancer (NSCLC) not directly touching the pleura on CT have still been reported to exhibit PI. Pleural tags (PT), defined as linear opacities extending from tumors to the pleura (7), are potentially indicative of PI (9–12). Additionally, PT with pleural indentation and PT with pleural thickening or soft tissue opacities, which do not conclusively indicate the absence of direct microscopic pleural contact (13), have been found to increase the likelihood of PI (8–10, 14). Hsu, J.-S. et al. found that the absence of PT was accompanied by the absence of PI in NSCLCs without the appearance of pleural contact on CT, while PT with soft tissue opacities at the pleural end was moderately associated with PI, with sensitivity and specificity of predicting PI at 36.4% and 92.8%, respectively (9). The low sensitivity might be due to the complex underlying pathology of PT, consisting of the pleura arranged in parallel, thickened interlobular septa, and fibrosis (15). Thus, CT parameters capable of distinguishing the pleura from other pathological subtypes of PT may indicate PI and warrant further exploration.

Tumor invasiveness has been reported to increase the occurrence of PI in NSCLC with pleural contact. Factors such as larger tumor size (7, 8, 16), solid density (7), spiculation sign (5, 17), and others have been associated with more frequent PI in various studies. Moreover, iodine uptake, derived from dual-energy CT imaging, correlates with tumor angiogenesis and invasiveness (18, 19). However, the impact of iodine uptake on PI in NSCLC with PT remains uncertain.

In this study, we aimed to preoperatively predict PI in ACCs with PT by identifying CT imaging parameters suggestive of PT consisting of the pleura and of tumor invasiveness through multivariate regression analysis.

## Methods

### Participants

This retrospective cohort study received approval from The Ethics Committee of our institution (Approval number: xmzsyyky-2023-119), and the requirement for patient informed consent was waived. No information relating to an identified or identifiable natural person were provided. All procedures were performed in compliance with Declaration of Helsinki, and institutional guidelines.

**Figure 1:**
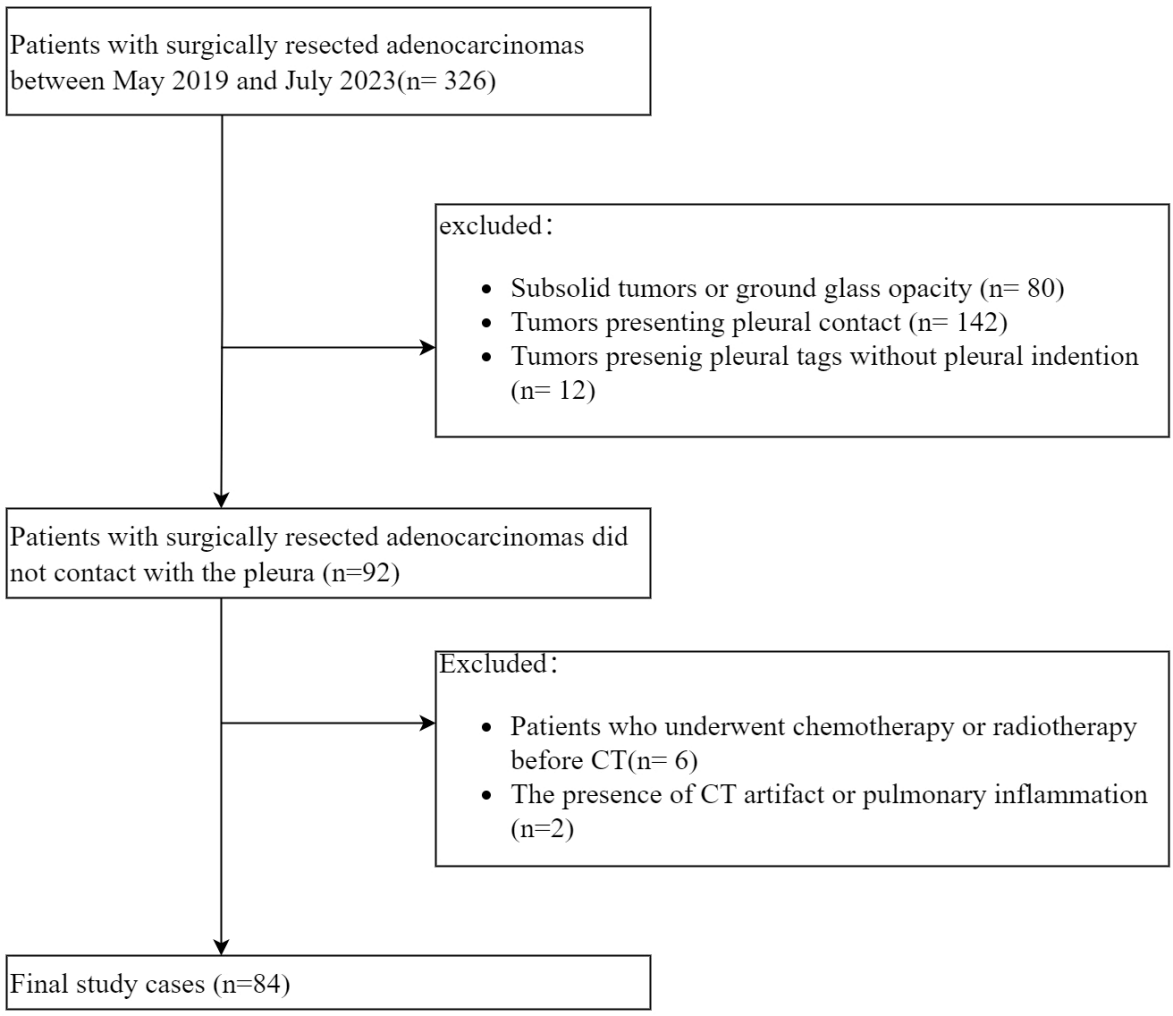
Flow chart of patient selection.

A total of 326 consecutive patients with ACCs underwent resection at our hospital between May 2019 and July 2023. The inclusion criteria included (a) tumors exhibiting PT with pleural indentation; (b) solid tumors, excluding those with ground glass opacity. The exclusion criteria were (a) tumors presenting pleural contact or PT without pleural indentation; (b) patients who underwent chemotherapy or radiotherapy prior to CT; (c) the presence of CT artifact or pulmonary inflammation potentially affecting CT imaging analysis. Ultimately, 84 patients were enrolled in the final cohort (**Figure 1**).

### CT imaging

All patients underwent enhanced CT scanning (IQON, Philips Healthcare, Germany) following a standardized protocol before tumor resection. Each patient received approximately 1.0 ml/kg of iodinated contrast agent (Iopromide, 300 mg/ml, Guangzhou, China) with the injection rate of 3 mL/s. The arterial phase scan commenced when the attenuation of the descending aorta (at the tracheal bifurcation level) reached 100 HU, followed by the venous phase scan approximately 25 seconds after the arterial phase scan concluded. All patients were asked to take a deep breath and hold it before each scanning. The scanning range was set as follows: from the thoracic inlet to the adrenal glands. CT scan settings were listed below: slice thickness, 2 mm; increment, 2 mm; collimation, 64 × 0.625 mm; matrix, 512 × 512; pitch, 0.891; rotation time, 0.5 s; tube voltage, 120 kVp. An automatic exposure control technique, DoseRight Index (DRI), was set as 17 to manage the radiation dose received by participants. Venous phase CT imaging raw data were extracted and transferred to a post-processing workstation, followed by the automatic generation of the multiple planar reconstruction images and iodine uptake images with a slice thickness of 0.625 mm.

**Figure 2:**
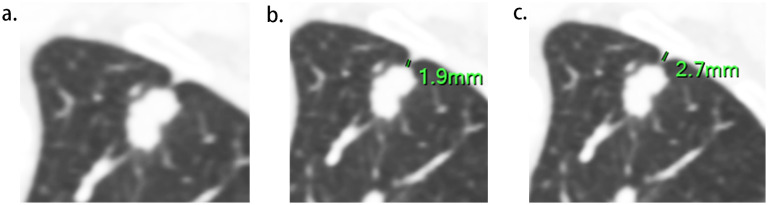
Measurement of LPT (the length of Pleural tag) and DPI (depth of pleural indentation). **(A)**, The slice with the shortest LPT. **(B)**, LPT, measured from the tumor margin to the retracted pleura. **(C)**, DPI, measured by the distance of pleural indentation.

**Figure 3:**
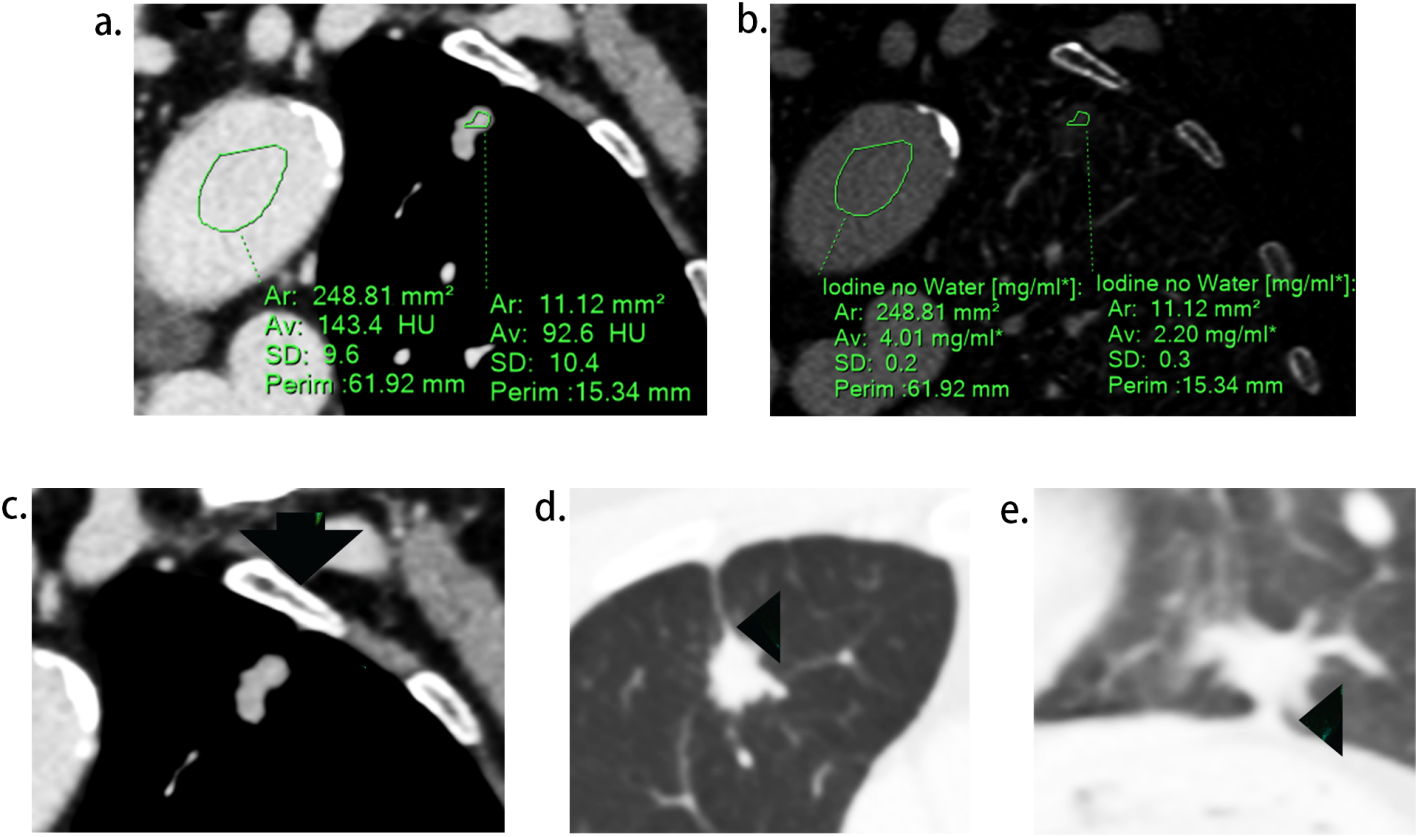
Representative images during CT imaging evaluation. **(A)**, regions of interest (ROI) delineated in lesion and aorta respectively. **(B)**, iodine concentration of the lesion and aorta on the iodine map. **(C)**, soft tissue opacity at the pleural end of the PT (arrow). **(D)**, tumor protrusion (arrow head), defined as triangular opacities extending from the tumor margins to PT. **(E)**, cord-like PT (arrow head), defined as a PT thicker than 2 mm.

**Figure 4:**
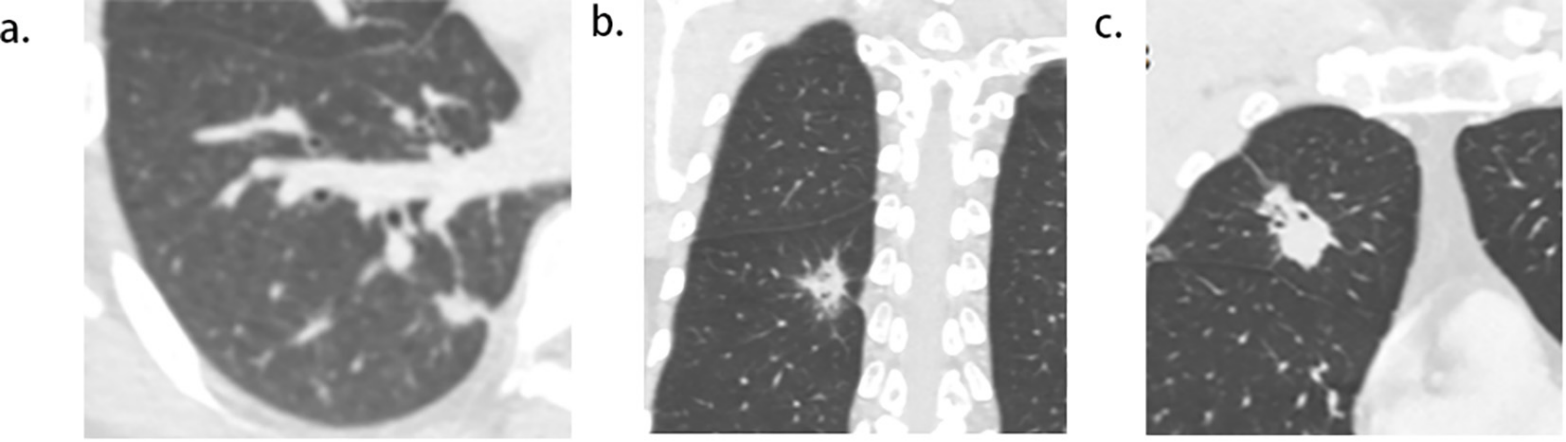
Pictogram of multiple pleural tags (PTs) to multiple types of pleura. **(A)**, single PT to single pleura (costal pleura in this case). **(B)**,multiple PTs to single pleura (costal pleura in this case). **(C)**, multiple PTs to multiple pleura (costal pleura and fissure were involved in this case).

Two radiologists (experienced in diagnosing lung cancers and blind to the status of PI), independently reviewed all CT images on the workstation. We selected the shortest distance from the tumor margin to the pleura as a criterion to identify the target pleural tag from multiple instances. This approach aligns with standard practices in pathology departments and reduces discrepancies between target PT on CT and the actual pleural sites, as pathologists often select slices where lesions abut or are close to the pleura to evaluate the status of pleural invasion. The length of PT (LPT), defined as the shortest distance from the tumor edge to the retracted pleura, was measured on lung windows (window level: -600 HU, window width: 1600 HU) (**Figures 2A, B**), and PT with shorter LPT was selected by the observers as a priority for further evaluation. On the slice with the minimum LPT, they assessed the depth of pleural indentation (DPI) (**Figure 2C**), iodine concentration (IC), the presence of pleural thickening, tumor protrusion and the presence of cord-like PT. A region of interest (ROI) was drawn freehand to cover the ipsilateral half of the lesion on mediastinal windows (window level: 40 HU, window width: 350 HU) (**Figure 3A**), avoiding areas of calcification, obvious necrosis, and large vessels. Lesion IC in the ROI and IC in the center of the aorta was automatically calculated on iodine uptake images (**Figure 3B**). Normalized IC (NIC) was calculated as the ratio of lesion IC to aorta IC. The presence of pleural thickening was diagnosed when the pleura was thicker than adjacent normal pleura or when soft tissue opacity was observed at the pleural end of PT on mediastinal windows (**Figure 3C**). Tumor protrusion was identified by wedge-shaped or triangular opacities extending from the tumor margins to the PT (**Figure 3D**), while cord-like PT was defined as a PT thicker than 2 mm (**Figure 3E**). The ratio of LPT to DPI was also calculated. Multiple PTs to multiple types of pleura was defined when the tumor with multiple PTs involving more than one types of pleura (costal, fissure, mediastinum, diaphragm) (20)(**Figures 4A–C**).

Tumor size was measured on the slice displaying the lesion’s maximum diameter on lung windows. The location, morphology (regular or lobulation), presence of bronchial obstruction, and vacuole sign were also documented. Bronchial obstruction was noted when bronchi were disrupted at the tumor’s proximal margin. The vacuole sign was identified when a small (less than 5 mm) round lucency or tubular lucency was observed within the tumor.

The same threshold of the attenuation to start contrast scan, consistent thin slice thickness and CT window parameters settings during images evaluation, were both important factors to keep the reproducibility of the study’s results. The discussion between observers was conducted to reach a consensus when any disagreements on CT image evaluations occurred.

### Pathologic evaluation

The status of PI was determined from the pathological records of resected specimens. Pathological slices were routinely stained with hematoxylin-eosin post-surgery, and Verhoeff–Van Gieson staining was applied as necessary (56 cases in this study). An experienced pathologist, re-sliced and reviewed the debatable cases to align with the target PT on CT as accurately as possible, ultimately categorizing all cases into two groups: PI- group and PI+ group.

### Statistical analysis

The normality of distribution for continuous variables was assessed using the Kolmogorov−Smirnov test. Normally distributed data are presented as means ± standard deviations, while non-normally distributed data are reported as medians and interquartile ranges (IQRs). Categorical variables are expressed as numbers and percentages. The Kolmogorov−Smirnov test for non-normally distributed continuous data and the independent sample t-test for normally distributed continuous data were used to compare CT features between ACCs with PI+ and PI-, while the χ2 test or Fisher’s exact test was applied to categorical data. Variables that were statistically significant in univariate regression analysis were introduced into a binomial logistic regression analysis to identify the independent predictive factors, utilizing a forward selection model (likelihood ratio). To assess predictive efficiency, analyses of receiver operating characteristic curves and accuracy analyses were conducted. All statistical analyses were performed using the SPSS statistical software package (version R26; IBM Corp). A two-tailed *p* value < 0.05 was deemed statistically significant.

## Results

### Patient details

A total of 84 patients, pathologically diagnosed with solid ACCs with PT on CT, were enrolled in this study. The median age of the participants was 62.0 ± 13.8 years, comprising 45 females and 39 males. Among them, 24 (28.6%) were smokers or ex-smokers. The average tumor size was 20.4 mm. Pathological PI was observed in 32 out of 84 (38.0%) patients. Additionally, 3 of 6 patients with malignancy history had received chemotherapy before. Moreover, it’s worth noting that 2 cases from 7 patients who received sub-lobectomy proved to have PI after surgery, in which status T stage upgrading and lobectomy were mandatory. Details of the patients are provided in **Table 1**.

**Table 1:** Patient data.

Variables	Datum
Total cases	84
Age, median ± IQR^a^, years	62.0 ± 13.8
Gender	
Man	39
Women	45
Tumor size, mean ± SD^b^, mm	20.4 ± 9.5
Smoking history	
No	60
Yes	24
History of malignancy	
No	78
Yes	6
Tumor location	
Left upper lobe	24
Left lower lobe	9
Right upper lobe	25
Right middle lobe	4
Right lower lobe	22
Pleural invasion	
No	52
Yes	32
Lobectomy	77
Sub-lobectomy	7

a, Interquartile range.

b, Standard deviation.

### The comparison of CT features between PI+ and PI- group

The PI+ group showed a larger tumor size (26.1 mm vs. 16.8 mm, *p* = 0.000)compared to the PI- group. No significant differences were observed regarding morphology, bronchial obstruction, and vacuole sign between the two groups (all *p* > 0.05) (**Table 2**).

**Table 2:** Comparison of CT features between adenocarcinomas with or without pleural invasion.

Variables	Total, N(%)	Pleural invasion		P value
		Positive (%)	Negative (%)	
Tumor size				0.000*
Mean ± SD^a^, mm	20.4 ± 9.5	26.1 ± 16.1	16.8 ± 5.6	
Morphology				0.12
regular	6(7.6)	0 (0)	6(12)	
Lobulated	78(92.3)	32(100)	46(88)	
Bronchial obstruction				0.20
No	71(84.5)	25(78.1)	46(88.5)	
Yes	13(15.5)	7(21.9)	6(11.5)	
Vacuole sign				0.48
No	55(65.5)	19(59.3)	36(69.2)	
Yes	29(34.5)	13(40.7)	16(30.8)	
Pleural tag				
LPT^b^				0.21
Median ± IQR^c^, mm	1.6 ± 1.5	1.5 ± 1.4	1.8 ± 2.3	
DPI^d^				0.41
Median ± IQR, mm	2.5 ± 1.8	2.5 ± 2.8	2.2 ± 1.7	
Ratio^e^	0.59 ± 0.83	0.50 ± 0.5	0.69 ± 1.2	0.07
Cord-like pleural tag				0.47
No	59(70.2)	21(65.6)	38(73.1)	
Yes	25(29.8)	11(34.4)	14(26.9)	
Pleural thickening				0.50
No	54(61.9)	22(68.8)	32(61.5)	
Yes	30(38.1)	10(31.2)	20(38.5)	
Multiple pleura^f^				0.000
No Yes	42(50.0) 42(50.0)	8(25.0) 24(75.0)	34(65.4) 18(34.6)	
Tumor protrusion				0.62
No	29(34.5)	10(31.3)	19(36.5)	
Yes	55(65.5)	22(68.7)	33(63.5)	
Iodine uptake				
IC^g^, mean ± SD, mg/ml	1.60 ± 0.46	1.66 ± 0.62	1.57 ± 0.33	0.47
NIC^h^, mean ± SD	0.50 ± 0.17	0.48 ± 0.18	0.50 ± 0.17	0.59

a, Standard deviation; b, the length of pleural tag; c, Interquartile range; d, the depth of pleural indention; e, the ratio of LPT to DPI; f, multiple pleural tags to multiple types of the pleura; g, Iodine concentration; h, normalized iodine concentration.

*, P < 0.05.

The median LPT was 1.6 ± 1.5 mm. The PI+ group showed higher presence of multiple PTs to multiple types of pleura (57.1% vs. 19.0%, p=0.000) compared to the PI- group. No significant differences were found regarding LPT, DPI, ratio, cord-like PT, pleural thickening, and tumor protrusion between the PI+ group and PI- group (all *p* > 0.05) (**Table 2**).

The mean IC and NIC were 1.60 mg/ml and 0.50, respectively. There were no significant differences in IC and NIC between the PI+ group and PI- group (**Table 2**).

Tumor size (OR 1.17, 95% CI 1.07-1.28, *p* = 0.001), LPT (OR 0.46, 95% CI 0.23-0.92, *p* = 0.028) and multiple PTs to multiple types of pleura (OR 3.58, 95% CI 1.13-11.20, *p* = 0.03) were ultimately included in the binomial logistic regression model (**Table 3**).

**Table 3:** Univariate and multivariate regression analysis for pleural invasion.

variables	Univariate analysis OR^a^ (95% CI^b^)	P value	multivariate analysis OR (95% CI)	P value
Tumor size	1.12(1.05-1.20)	0.001	1.17(1.07-1.28)	0.001
LPT^c^	0.69(0.46-1.01)	0.06	0.4(0.23-0.92)	0.028
DPI^d^	1.30(1.00-1.69)	0.06		
Ratio**^e^**	0.32(0.12-0.86)	0.02		
Multiple pleura^f^	5.67(2.12-15.15)	0.001	3.58(1.13-11.20)	0.03

a, odds ratio; b, confidence interval; c, the length of pleural tag; d, the depth of pleural indention; e, the ratio of LPT to DPI; f, multiple pleural tags to multiple types of the pleura.

### Predictive efficiency analysis for PI

Predicting PI using tumor size with the optimal cutoff value of 22.8 mm resulted in a high specificity of 88.5%, with sensitivity and accuracy being 53.1%and 75.0%, respectively. Compared to tumor size, LPT demonstrated a higher sensitivity (96.9%) and lower specificity (25.0%) when an LPT < 3 mm was used as the diagnostic criterion. In our study, one patients in subgroup with tumor size <10mm and 11 patients in subgroup with tumor size between 10mm and 20mm were pathologically proved to be pleural invasion positive, all of who showed LPT<3mm. As for multiple PTs to multiple types of pleura, the predictive sensitivity and specificity were 75.0%, 65.4%, respectively. The areas under the curve (AUC) derived from ROC curves and accuracy was marginally elevated by combining the three CT features, resulting in a sensitivity, specificity, accuracy of 50%, 94.2%, 77.4%, respectively (**Table 4**).

**Table 4:** Diagnostic efficiency for pleural invasion.

Predictors	Sensitivity	Specificity	accuracy	AUC^a^
Tumor size>22.8 mm	53.1%	88.5%	75.0%	0.76
LPT^b^ <3 mm	96.9%	25.0%	52.4%	0.62
Multiple pleura	75.0%	65.4%	69.0%	0.70
Combination^c^	50.0%	94.2%	77.4%	0.84

a, the area under curve; b, the length of pleural tag; c, the combination of three CT features.

## Discussion

In this investigation, we discovered that tumor size, LPT and multiple PTs to multiple types of pleura were effective independent predictors of PI in ACCs with PT. High specificity was achieved for PI prediction with tumor sizes > 22.8 mm and higher sensitivity with LPT < 3 mm, at 88.5% and 96.9%, respectively. Furthermore, the combination of these three parameters slightly improved the predictive efficiency, yielding a higher accuracy and AUC.

**Figure 5:**
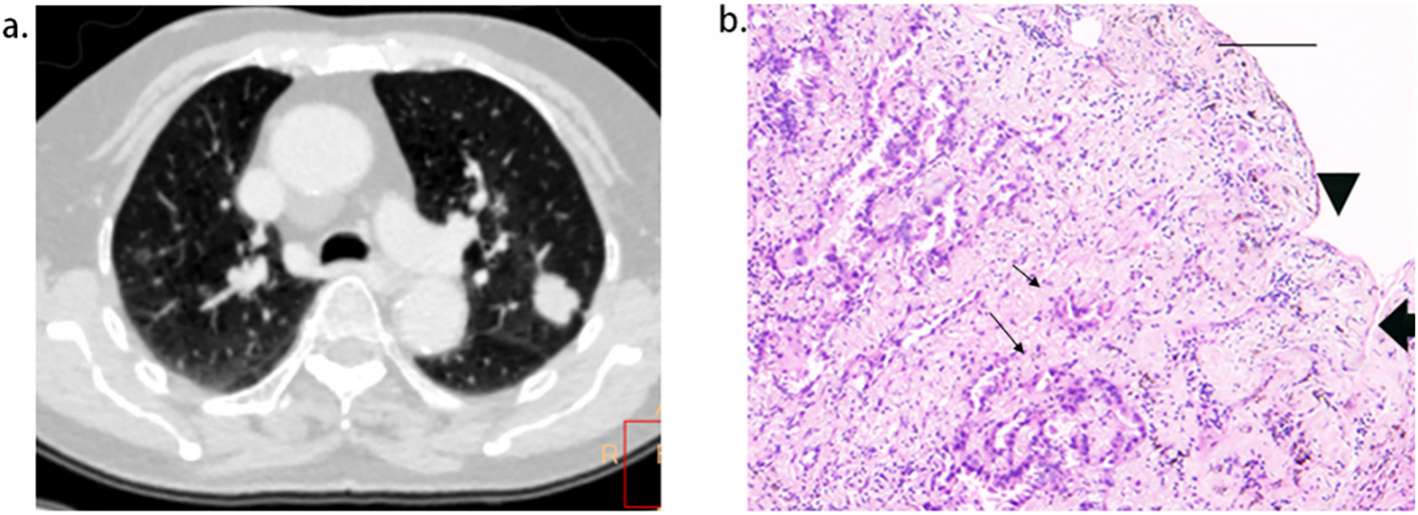
Short length of pleural tag (PT) was indicative of PT consisting of the pleura. **(A)**, adenocarcinoma presenting PT with pleural indentation. **(B)**, Scale bar = 2 mm; hematoxylin-eosin stain; magnification, X 40; Microscopic pleural indentation (arrow head) and the pleura arranged in parallel (thick arrow); Tumor cells infiltrated the pleura (thin arrow).

Tumor size was notably larger in cases with PI than in those without (26.1 mm vs. 16.8 mm), establishing it as a statistical predictor of PI in ACCs with PT. Tumor size is a well-known indicator of tumor invasiveness, as larger tumors are often associated with more invasive pathological subtypes (19), lower differentiation (21), irregular shape (22), and more frequent lymph node metastases (23). Several studies have highlighted the significance of tumor size in predicting PI in ACCs (8, 16, 24). Shi, J. et al.’s findings that larger tumor sizes are linked to PI in ACCs with PT align with our results (7). However, Hsu, J. et al.’s study, which did not find a statistical correlation between tumor size and PI in ACCs with PT (9), contrasts with our findings. This discrepancy may stem from differences in the study populations. Unlike their study, which included all types of PT, ours focused exclusively on PT with pleural indentation, typically associated with larger tumor sizes (22). Our paper also identified LPT as an independent predictor of PI in ACCs with PT. LPT, the shortest length of PT, relates closely to DLP (the distance from the lesion to the pleura). Deng, H.-Y., et al. demonstrated that NSCLCs with a DLP < 10mm are more likely to exhibit PI than those with a DLP > 10 mm (25), while Qi, L., et al. found similar outcomes for DLP < 5 mm (10). However, these studies primarily focused on tumors in direct contact with the pleura, and to the best of our knowledge, few have concentrated on tumors without pleural contact. Papillary/acinar subtype ACCs have been observed to have a smaller distance from the invasive component to the pleura than the alveoli/lepidic subtype (26), suggesting that a short DLP is associated with tumor invasiveness. Additionally, the resistance of adjacent inflated lung tissues gradually limits the movement of the retracted pleura toward tumors (13, 27, 28), resulting in short, parallel arrangements of pleura on the tumor side (**Figure 5**). Conversely, the extension of interlobular septa and fibrosis faces fewer restrictions. Therefore, a small LPT indicates that PT primarily consists of pleura and is partially associated with tumor invasiveness. An LPT < 3mm demonstrated a high sensitivity of 96.9% for predicting PI, supporting its utility in predetermining PI in this study. Additionally, our study found that multiple PTs to multiple types of pleura was independent predictor for PI, correspondent with Q, S., et al.’s recent work (20), in which the sensitivity and specificity were 11%, 98%, respectively. The underline explanation for this statistical significance was not merely more pleural contact sites increasing the possibility of pleural invasion, because previous investigations did not reveal multiple PTs involving single pleura correlated with PI (13, 27). A higher invasiveness to infiltrate in more directions maybe the additive interpretation. The combined use of tumor size, LPT, and multiple PTs to multiple types of pleura slightly improved predictive efficiency for PI, achieving higher accuracy and AUC. However, the low sensitivity and moderate accuracy of prediction still existed and align with previous reports (9, 29).

Contrary to initial expectations, cord-like PT, hypothesized to have a longer pleural contact than linear PT and potentially increase PI occurrence, showed no correlation with PI in ACCs with PT. Similar findings were reported by Shi, J., et al. and Hsu, J.-S., et al. (8, 9), suggesting that the contact length was insufficient to increase PI occurrence. Ebara, K., et al. proposed that an interface length < 4.1 mm might exclude PI in lung cancers with pleural contact (30), supporting this notion. Iodine concentration (IC), a novel parameter derived from spectral CT, reflects the iodine uptake of lesions. IC at the venous phase is positively related to tumor angiogenesis and histological grade, indicators of tumor invasiveness. However, our paper found no significant difference in venous phase IC between tumors with and without PI, aligning with previous findings (5, 31).

There are several limitations to this study. The number of patients included in this investigation was not large enough after rigorous screening of the participant, posing statistical challenges for working out valuable CT features for independently predicting PI, also limiting the generalizability and robustness of the results. Further research with a larger sample size is needed to confirm the conclusions of this study. Second, the study was retrospective, increasing the risk of selection bias and limiting the efficiency of statistical analysis, hence prospective studies should be arranged in the future to validate these findings. Third, a high predictive sensitivity has been achieved by LPT <3mm, but merely moderate sensitivity was worked out combining three CT features to predict PI, indicating that the predictive model might not capture all cases of pleural invasion. Future work focusing on new radiologic parameters (such as MR indicators) or other biomarkers should be considered.

In conclusion, this paper determined that LPT is indicative of PT primarily consisting of pleura and effective for predetermining PI in ACCs with PT. Additionally, the combination of tumor size, LPT, and multiple PTs to multiple types of pleura improved the predictive performance for preoperatively identifying PI, achieving high specificity and moderate accuracy, but the low sensitivity and moderate accuracy of prediction still need to be improved.

## Data Availability

The datasets presented in this study can be found in online repositories. The names of the repository/repositories and accession number(s) can be found in the article/supplementary material.
